# Cloacal and Ocular Microbiota of the Endangered Australian Northern Quoll

**DOI:** 10.3390/microorganisms6030068

**Published:** 2018-07-12

**Authors:** Catherine Burke, Delaney Burnard, Adam Polkinghorne, Jonathan Webb, Wilhelmina M. Huston

**Affiliations:** 1 School of Life Sciences, Faculty of Science, University of Technology Sydney, NSW 2007, Australia; Catherine.Burke@uts.edu.au (C.B.); Jonathan.Webb@uts.edu.au (J.W.); 2 Centre for Animal Health Innovation, Faculty of Science, Health, Education and Engineering, University of the Sunshine Coast, Maroochydore, QLD 4558, Australia; Delaney.Burnard@research.usc.edu.au (D.B.); apolking@usc.edu.au (A.P.)

**Keywords:** northern quoll, microbiota, cloaca, marsupial, 16S rRNA gene sequencing

## Abstract

The Australian northern quoll is an important predatory marsupial carnivore that is currently endangered due to inappropriate fire regimes, predation, and the spread of invasive cane toads. The microbiota of Australian marsupials has not been extensively studied, but is thought to play a role in their health. This study provides an initial characterization of the cloacal microbiota of the northern quoll, as well as other marsupials including possums and kangaroos which were opportunistically sampled. The northern quoll cloaca microbiota was dominated by *Enterococcus* and *Lactobacillus* and had a relatively high proportion of members of the Proteobacteria phylum, which has been observed in other carnivorous marsupials. The diversity and structure of the microbiota was not influenced by presence of *Chlamydiales* which are intracellular bacteria and potential pathogens. The microbiota of the other marsupials was quite varied, which may be related to their health status. Characterization of the northern quoll microbiota will help to better understand the biology of this endangered animal.

## 1. Introduction

Predators exert strong effects on ecosystem community structure by influencing the behavior and demography of the species on which they prey [1,2]. The northern quoll (*Dasyurus hallucatus*) is the largest surviving predatory marsupial carnivore in Northern Australia [3] and is currently listed as an endangered species [4]. Northern quolls historically ranged uninterrupted from South East Queensland to the Kimberley and Pilbara regions in Western Australia, but since European settlement have seen a reduction of 75% [3]. Their decline has been attributed to factors such as land management practices and increased risk of predation [5,6]. The spread of the invasive, highly toxic cane toad *Rhinella marina* is considered one of the greatest threats to the northern quoll, with their arrival associated with population crashes and local extinctions in Kakadu National Park [7]. 

In an effort to preserve the species, in 2003, wildlife authorities translocated 64 quolls to Astell and Pobassoo Islands. These islands are located north of the Northern Territory, and are free from cane toads and mammalian predators of the northern quoll, such as feral cats and dingoes. These island populations were monitored to assess population growth, genetic diversity, and condition, via bait and capture methods [8]. It was hoped that control of cane toad populations, in conjunction with new methods for training quolls not to eat cane toads, [9] would allow these populations to be used as a source for re-population of mainland Australia in the future [10].

In 2016, researchers captured quolls from Astell Island for a breeding program at the Territory Wildlife Park. Progeny of those quolls were subsequently trained to avoid eating cane toads, and were reintroduced to Kakadu National Park [11]. The quolls were taken to the Territory Wildlife Park, which provided an opportunity to characterise and study the quoll microbiota, which has not been studied previously. Host associated microbial communities are known to influence the health and wellbeing of their hosts [12], and dysbiotic microbiota have been associated with a wide range of diseases in humans including irritable bowel disease [13] and bacterial vaginosis [14]. Links between host health and gut microbiota also have been observed in livestock [15,16] and wild animals [17,18]. A limited number of studies have explored the microbiota of Australian marsupials, including wombats [19], Tasmanian Devils [20], and koalas [21,22], with an emphasis on improved understanding of marsupial biology and conservation management. Cheng et al. [20] found significant differences in captive versus wild populations of the Tasmanian Devil, while Vidgen et al. [22] found correlations between the koala ocular and urogenital microbiota and *Chlamydia pecorum* infection, a major infectious disease threat to this iconic arboreal marsupial [20]. 

In order to provide an initial overview of the northern quoll microbiota, we characterised the microbial community from the cloaca (*n* = 27) and ocular cavity (*n* = 7) in 31 individual quolls. Samples from a range of Australian marsupials (three Arnhem rock rats, three spotted tail quolls, seven possums, one wallaby, and two kangaroos) were collected and sequenced separately and their microbiota used for a qualitative comparison. Chlamydial detection was previously established in this sample set, revealing a range of previously characterized and novel *Chlamydiales* genotypes in quolls and other non-koala marsupials [21]. *Chlamydia pecorum* has previously been correlated with changes to microbial community structure [22]. To determine whether other members of this order have similar effects on the microbiota, we also analysed microbial diversity in the context of *Chlamydiales* detection.

## 2. Method

### 2.1. Northern Quolls Sample Collection and Processing

#### 2.1.1. Sample Collection

Samples were collected from 34 northern quolls (*Dasyurus hallucatus*) and three Arnhem rock rats (*Zyzomys mainii*) from the Northern Territory. Animals were trapped at sites located on Astell Island (11°53.129′ S, 136°25.497′ E) and Kakadu National Park (12°25.884′ S, 132°57.121′ E) in February and May 2016, respectively. At each location, wire mesh cage traps (single door Chipmunk/rat traps, 16 × 5 × 5 inches, Tomahawk Live Trap, Hazelhurst, WI, USA) baited with a mixture of peanut butter, honey, and oats, were placed in suitable locations (under ledges, inside crevices) in the late afternoon. Traps were checked within 2 h of sunrise the following morning, and the sex, mass, reproductive status, and microchip number of animals captured inside traps was recorded. At Kakadu, cloacal and ocular samples were taken from three quolls and three rock rats using swabs (Copan Floqswabs, Brescia, Italy) in the field, and samples were frozen within 3 h. On Astell Island, quolls were transported to the Territory Wildlife Park as part of a captive breeding program. Upon arrival to the wildlife park, each quoll underwent a thorough examination from a veterinarian. Cloacal and ocular samples were taken from 31 quolls during the examination, and samples were frozen within 20 min. All samples were collected with Copan floqswabs (519C) and stored in 1 mL of 1 × TE. Samples were transported to the University of Technology Sydney for further processing. All work was done in accordance with the University of Technology Sydney Animal ethics approval number ACEC 2105000175.

#### 2.1.2. DNA Extraction

Swabs were thawed on ice, vortexed, and the TE suspensions were processed for DNA using a GenElute bacterial gDNA kit (Sigma-Aldrich, Sydney, Australia) as per the manufacturer’s instructions. DNA concentration for each sample was measured using a Qubit and a high sensitivity double stranded DNA fluorescence assay (ThermoFisher Scientific, Waltham, MA, USA).

#### 2.1.3. 16S rRNA Gene Library Preparation and Sequencing

Samples were prepared for 16S rRNA gene sequencing using a two stage PCR protocol. The first PCR used primers that target the bacterial 16S rRNA gene (V3-V4 region), flanked by partial Illumina adaptors at the 5′ end (Table 1). Two ng of DNA was used as template where possible, otherwise the maximum volume of sample permitted in the PCR reaction was used (20 μL in a 50 μL reaction). After 20 cycles of PCR, the reactions were cleaned and concentrated (Axygen PCR-mag, FisherScientific, Waltham, MA, USA) and a second round of amplification was performed for 10 cycles with primers containing Illumina adapters and sample indexes of eight nucleotides. Kapa HiFi ready master mix (Roche) was used for all PCR reactions. PCR reactions were cleaned to remove excess primers (Axygen PCR-mag), and quantified using a Qubit fluorescence assay. See supplementary methods for a detailed PCR protocol (Supplementary Materials S1).

Sequencing controls included DNA extraction process negatives, PCR negatives, and two mock community standards, the even mock community from the Human Microbiome Project (https://www.hmpdacc.org/HMMC/) which is a defined mixture of DNA from of 21 bacterial and one yeast species, and the Zymobiomics microbial community DNA standard (Zymo Research, D6305, Irvine, CA, USA) which contains eight bacterial and two yeast genomes.

PCRs from each sample were pooled equally by molarity. Where the DNA concentration was too low to be detected (i.e., negative controls), the median sample volume was used to add that sample to the pool. The pooled sample was assessed for quality and quantity on a Bioanalyser using a high sensitivity DNA chip (Agilent, Santa Clara, CA, USA). The pooled sample was loaded onto a MiSeq flow-cell at 6pM with 5% PhiX control and sequenced for paired ends using a V2 500 cycle kit. Sequencing was carried out at the University of Technology Sydney.

### 2.2. Other Marsupial Sample Collection and Processing

#### 2.2.1. Sample Collection and DNA Extraction

Urogenital swab samples were opportunistically collected from marsupials presenting at collaborating veterinarian or wildlife centres in Queensland and New South Wales, as previously reported [21]. Samples were collected from four ringtail possums (*Pseudocheirus peregrinus*), two brushtail possums (*Trichosurus vulpecula*), one short eared possum (*Trichosurus caninus*), two Eastern Grey kangaroos (*Macropus giganteus*), two spotted tail quolls (*Dasyurus maculatus*), one long nosed bandicoot (*Perameles nasuta*), and one swamp wallaby (*Wallabia bicolor*). Samples were collected in accordance with animal ethics number is ANS1539, awarded by the University of the Sunshine Coast Animal Ethics Committee. Swabs were previously processed for DNA using a QIAamp DNA mini kit (Qiagen) according to the manufacturer’s instructions. In the current study, DNA was quantified using a Quant-iT dsDNA High Sensitivity kit (ThermoFisher Scientific, Waltham, MA, USA). Sample concentrations were normalised to between 5 and 10 ng/μL by diluting in molecular water.

#### 2.2.2. 16S rRNA Gene Library Preparation and Sequencing

Sequencing libraries were generated using the protocol outlined in Fadrosh et al. [23]. In brief, a one-step PCR protocol is used with primers that include regions complementary to the V3 (319F) or V4 (806R) 16S rRNA gene, sample indexes of 12 nucleotides, and Illumina adapter sequences. Approximately 25 ng of template DNA was added to each reaction, using the Kapa HiFi hot-start ready mix with a total reaction volume of 25 μL, and 30 cycles. PCR products were cleaned and the concentration normalised with a SequelPrep Normalisation plate kit (ThermoFisher Scientific, Waltham, MA, USA). Equal volumes of each sample were pooled, then concentrated using a DNA Clean and Concentrator kit (Zymo Research). The pooled sample was quantified using a Qubit HS double stranded DNA assay (Thermo Fisher), then sent to the Ramaciotti Center for Genomics (University of New South Wales, Sydney, Australia) for paired end sequencing on a MiSeq with a V3 600 cycle kit.

#### 2.2.3. 16S rRNA Gene Sequence Analysis

The two data sets (marsupials and Northern Quolls) were initially processed separately for quality filtering and demultiplexing, due to the different adapter design. 

For the northern quolls data, raw sequencing reads were assessed for quality using FastQC [24]. Sequences were demultiplexed and quality filtered using Qiime v1.9.1 [25] and the default settings in the split_libraries_fastq.py command, such that the sequence headers were changed to reflect the name of the sample and sequence number. Primer and adapter sequence were removed using cutadapt [26]. Low sequence quality was observed for read 2 (median q score 27). Merged read 1 and read 2 sequences were less representative of the mock community than using read 1 alone (see supplemental information). As such, only read 1 (corresponding to the V4 region) was used for downstream analysis.

For sequences data from the other marsupials, paired end sequences were merged using PEAR [27], then demultiplexed in Qiime 1.9.1 using the split_libraries.fastq.py command with default settings. Primer and adapter sequence were removed using cutadapt.

Demultiplexed sequences from both data-sets were combined at this point. Sequences were aligned in the Mothur v1.39.5 [28] using align.seqs against a Mothur formatted SILVA non-redundant database (Release 128) accessed from the Mothur website (https://www.mothur.org/wiki/Silva_reference_files). Alignments were trimmed to global positions that captured the majority of the reads, and filtered such that only sequences covering the V4 region (between *Escherichia coli* positions 515 and 806) were retained, using the pcr.seqs command. Gaps were removed from the filtered alignment before OTU picking.

The trimmed and filtered sequences were clustered into operational taxonomic units (OTUs) at 97% similarity using the open_ref_otu_clustering.py workflow script in Qiime v1.9.1, which includes removing OTUs which do not align to the 16S rRNA gene or represented by a single sequence, and creating a phylogenetic tree from representative sequences of each OTU using FastTree 2 [29]. Chimeras were detected using identify_chimeric_seqs.py and the UCHIME method [30], and filtered from the OTU table using the filter_seqs.py command.

Taxonomy was assigned to representative sequences from each OTU with the SINA 1.3.1 alignment and classification tool [31] with the SILVA REF NR 99 ssu database (released 13 December 2017), accessed from the SILVA website (https://www.arb-silva.de/projects/ssu-ref-nr/). 

Downstream analyses were performed in R [32] using the Phyloseq package v1.22.3 [33]. Based on the coverage of negative controls, samples with less than 25,000 sequences were removed, and all samples were rarefied to 25,572 sequences. Shannon (alpha) diversity and weighted unifrac distances (beta-diversity) and ordination were calculated using the phyloseq and vegan v2.4.6 [34] packages. The Kruskal–Wallis test was used for statistical comparisons of Shannon diversity (base R stats package). PERMANOVA [35] as implemented in the adonis function of the vegan package was used to determine significant differences between groups based on weighted unifrac distances and ordination. Plots were generating using a combination of Phyloseq, dplyr v0.7.4 [36] and ggplot2 v2.2.1 [37], except for heatmaps which were generated using the Complex Heat Map v1.17.1 [38] and circlize v0.4.3 [39] packages.

## 3. Results

### 3.1. Sequencing Data

After multiple sequence alignment and trimming to global positions, sequence lengths ranged from 226 to 240 base pairs, with a median of 227. These sequences were used for open reference OTU clustering which produced 22,239 non-singleton OTUs.

Negative sequencing controls were included at each stage of sample collection and processing, including field collection, DNA extraction, and PCR. Negatives from the PCR stage had read depths of 47 and 1753 reads, while process and field negatives ranged between 7 and 12,000 reads. The most common contaminants from PCR negatives were assigned to the *Corynebacterium* (35.8%) and *Roseburia* (13.2%), while field collection negatives were dominated by *Bacteroides* (38.8%) and *Pseudomonas* (18.0%). DNA extraction negatives were dominated by *Streptotoccus* (20.2%) and *Delftia* (16.2%).

In preliminary data analysis, we performed ordination and clustering on all samples with at least 5000 sequences including negative controls. Forty-eight of 137 samples had less than 5000 reads. We observed eight samples with read coverage from approximately 9000 to 20,000 that clustered closely with the negative controls. To avoid samples with possibly high levels of contaminating sequences, only samples with at least twice the read coverage of the highest coverage of a negative control were used, i.e., only samples with at least 25,000 reads were retained for analysis. Of the northern quolls samples collected and processed (56 cloaca and 57 ocular), 34 samples had read depth above 25,000 (27 cloaca and 7 ocular) from 31 individual quolls. Sequence coverage for all other marsupial samples (from a separate sequencing run) ranged from 2 to 841,000. Thirty samples had coverage above 25,000, and these were included for downstream analyses, and were rarefied to 25,572 sequences per sample for subsequent analyses. Sequence data from both sequencing runs has been deposited in the NCBI SRA under BioProject accession number PRJNA473283.

### 3.2. Northern Quoll Taxonomic Summary

**Figure 1:**
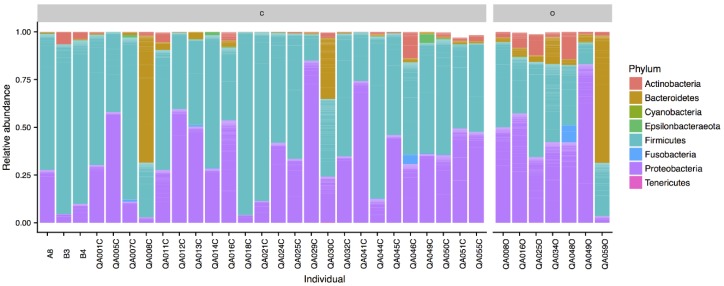
Phyla composition of Northern Quolls ocular (O) and cloaca (C) samples.

**Figure 2:**
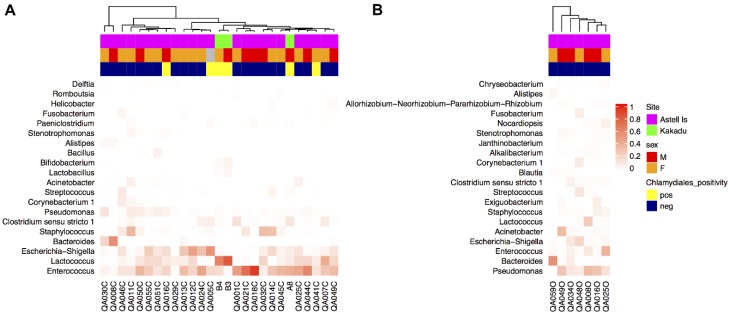
Heat map of the top 20 genera from (**A**) cloaca and (**B**) ocular samples from northern quolls. Clustering of samples was based on a weighted Unifrac distance matrix, and genera were arranged by relative abundance. Grey colouring in the top annotation bars indicates where metadata was not collected.

All taxa are reported in terms of their average relative abundance (percent ± standard deviation). Northern quoll microbiotas were dominated by the Firmicutes (58.1 ± 21.3% cloaca, 33.6 ± 12.8% ocular) and Proteobacteria (34.4 ± 21.3% cloaca, 44.6 ± 24.1% ocular) phyla (Figure 1), with smaller relative proportions of Bacteroidetes, Actinobacteria, and Fusobacteria. Within the Firmicutes, the majority of the sequences in both ocular and cloaca sites were assigned to the *Enterococcus* (27.3 ± 22.4% cloaca, 9.2 ± 13.3% ocular) and *Lactobacillus* genus (13.9 ± 19.0% cloaca, 4.7 ± 9.5% ocular). Within the Proteobacteria, the ocular sites were dominated by the *Pseudomonas* genus (19.1 ± 12.8%), while the cloaca contained *Escherichia* (11.5 ± 14.2%) with lower levels of *Pseudomonas* (2.6 ± 3.5%). The predominant genera for cloaca and ocular samples can be seen in Figure 2. Tests for significant differences between the two sites at genus level indicated that difference was primarily driven by a higher relative abundance of the *Enterococcus* genus in the cloaca (ANCOM, W = 47).

**Figure 3:**
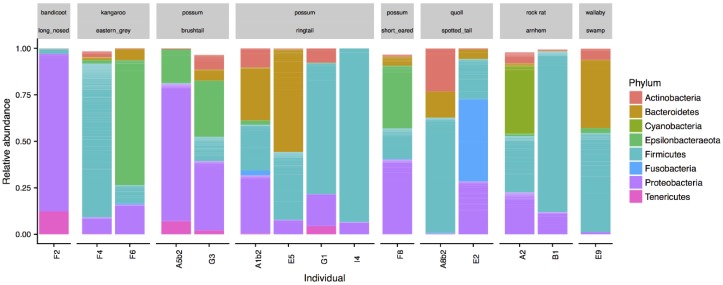
Phyla composition of cloaca microbiota from a range of marsupial species. Individual species are indicated in grey boxes.

The other marsupials studied included two spotted tail quolls, three possum species (two brushtails, four ringtails, and one short eared), two Eastern Grey kangaroos, one swamp wallaby, one long nosed bandicoot, and two Arnhem rock rats. All samples were collected from the cloaca. The composition of microbial communities from these marsupials was also dominated by the Proteobacteria (25.9 ± 25.0%) and Firmicutes (40.0 ± 31.9%) phyla, with lower relative abundances of Bacteroidetes (10.7 ± 16.6%), and Actinobacteria (4.5 ± 6.2%) (Figure 3). Different genera dominated different animals with obvious variation between individuals. For example, in the possums, *Escherichia* (50.4 ± 26.6%) and *Helicobacter* 24.1 ± 8.5%) dominated the brushtails, while two of the ringtails were dominated by *Gemella* (61.2 and 91.2%). One of the Eastern Grey kangaroos was dominated by *Campylobacter* (67.3%), the other by *Streptococcus* (13.9%) and the swamp wallaby by *Porphyromonas* (36.6%). 

### 3.3. Chlamydiales Detection is Not Correlated with Changes in the Northern Quoll Microbiota

*Chlamydiales* was previously detected in 19 of the 49 samples [40], which represented all marsupial species tested. In northern quolls, none of the ocular (*n* = 7) and six of the cloaca (*n* = 27) samples tested positive (as previously described) [40]. The remaining marsupial samples where *Chlamydiales* were detected included two spotted quolls, two rock rats, one wallaby, six possums, one kangaroo, and one bandicoot. We did not detect *Chlamydiales* 16S rRNA gene sequences in the data generated in this study from the same animals.

**Figure 4:**
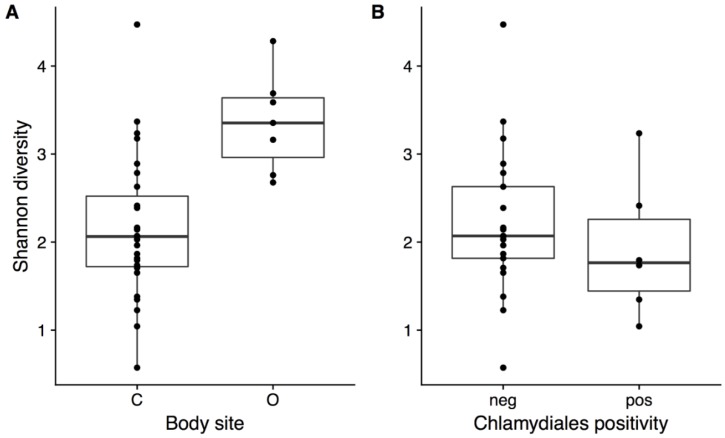
Shannon diversity in Northern Quolls for (**a**) cloaca vs ocular body site and (**b**) in the cloaca for samples which tested negative or positive for the presence of *Chlamydiales*. * indicates significant difference (*p* < 0.05, Kruskal–Wallis test).

**Figure 5:**
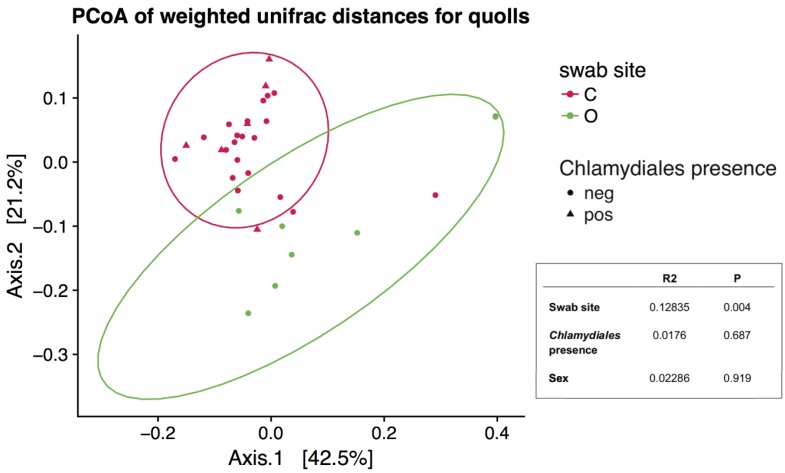
Principal Coordinates Analysis plot based on ordination of weighted Unifrac distances. Only northern quoll samples were included, ellipses represent 95% confidence intervals. Inset is the results from a PERMANOVA analysis, where R2 represents the effect size (from 0–1) and P is the *p* value.

The presence of *Chlamydiales* was not significantly correlated to diversity or microbial community composition. Shannon diversity was not significantly different in northern quolls regardless of presence of *Chlamydiales* (Figure 4b), but was significantly higher in ocular compared to all cloaca samples (Kruskal–Wallis, *p* = 0.002, Figure 4a). Community composition was not significantly different when considering *Chlamydiales* presence or sex, while body site was significant, accounting for 13% of variation (Figure 5).

## 4. Discussion

The northern quoll is an important marsupial predator that has suffered precipitous population declines across its geographic range. Significant range contractions and declines occurred prior to the arrival of toxic cane toads. Putative agents of decline include disease, altered fire regimes, and predation by feral cats and dingoes [3]. In order to better understand northern quoll biology, we have characterised the microbiota from the cloaca and/or ocular sites from 31 individual quolls, and 15 other opportunistically sampled marsupials.

The data from the northern quolls was collected separately to the other marsupials using different DNA extraction and PCR protocols, and from a separate sequencing run. We acknowledge that these differences will impact on the resulting community profiles [41,42] and as such we interpret this data with caution and have avoided making direct quantitative comparisons between the two. The data is also interpreted with an understanding of the limitations of 16S rRNA gene sequencing, as this data is compositional and does not represent absolute but rather relative abundances.

Only 7 of the 57 (12.3%) ocular samples collected were sequenced to a high enough read depth to be included in the analysis. A similar effect has been seen previously in koalas, where only 9.8% of ocular samples collected and processed were able to be included for analysis [22]. Given the low number of samples with adequate data, only limited conclusions can be drawn on the northern quoll ocular microbiome, and further studies with a larger sample size are needed. However, some trends consistent with other studies were observed, such as higher alpha diversity in ocular as compared to cloacal samples which has also been observed in koalas [21,22]. The *Pseudomonas* genus had the highest average relative abundance in the ocular microbiota, as has been previously reported in humans [43]. 

We observed similarities between the northern quoll cloaca microbiota and the gut microbiota in Tasmanian devils as reported by Cheng et al. [20]. Both animals are carnivorous marsupials from the family Dasyuridae. The northern quoll cloaca had a relatively high proportion of Proteobacteria (dominated by the Gammaproteobacteria class) (34.4 ± 21.3%) as did the previously reported Tasmanian devil fecal samples (18.6 ± 3.5%) [20]. Proteobacteria are present in much lower proportions (generally less than 10% relative abundance) in other mammal gut microbiotas [19,44]. Similarly, a low proportion of the Bacteroidetes phylum in the Tasmanian devil fecal microbiota (1.2 ± 0.6%) compared to other mammals (5–35%) [20] was also observed (4.5 ± 13.8%). These similarities may be related to both diet and phylogenetic relatedness, which has been shown to correlate with more similar microbiotas in mammals [44]. There were also distinguishing features observed in the northern quoll cloaca, such as the dominance of the *Enterococcus* genus (27.3 ± 22.4%). *Enterococcus* are commonly found in the mammalian gut microbiota, but usually in much lower proportions (for humans ~1%) [45]. None of the previous studies of marsupial microbiotas has reported such high proportions of *Enterococcus*. 

Cloacal swabs may represent both the gut and the urogenital microbiota, as swabs were collected from the cloacal opening which serves the digestive and reproductive/urinary tracts. *Lactobacillus* was the second highest genus in average proportion (13.9 ± 19.0%). This genus dominates the human female vaginal microbiota [46], and is a keystone taxa in this environment. While the koala urogenital tract was dominated by the Lactobacillales family, members of the *Lactobacillus* genus were only present at low proportions [22]. In the other marsupials studied here, only very low proportions of *Lactobacillus* were observed (0.00–5.3%). It is difficult to speculate why such high proportions were observed here compared to other marsupials since only limited studies have been carried out on marsupial urogenital microbiotas so far.

*Chlamydiales* are detected in a range of native animals in Australia, and while infection with *Chamydia* sp. is typically associated with disease the impact of *Chlamydia*-related bacteria (CRB) on host health is currently unclear (and those found in these animals may be commensal) [47]. *Chlamydiales* DNA was previously detected in six of the 27 northern quoll cloacal samples studied here [40], including all three from the Kakadu site. The sequences from the currently described samples were novel and could not be classified to the genus or species level [40]. We found no evidence that presence of *Chlamydiales* was associated with significant shifts in either alpha diversity or community composition, as has been previously described for koalas [22], while acknowledging that the small number of positive samples gives limited statistical power. We did not detect *Chlamydiales* 16S rRNA gene sequences in the data generated here from the same animals, which may be due to differences in primer specificity. The significance of their presence in the quoll population is unclear, and it would be interesting to determine if there is a higher prevalence in predator rich mainland compared to the predator free island populations.

The cloacal microbiotas of the other marsupials collected were quite varied (Figure 5). Given that these samples were collected opportunistically from animals brought to veterinary clinics for medical treatment, it is likely that they were unwell and the composition of their cloacal microbiotas may not be representative of that of healthy animals, as is often observed in humans [12]. Additionally, we have a very limited sample size for each species represented here. However, this is the first reported microbiota data for many of these species, highlighting the lack of knowledge of the microbiota of Australian native species. No previous studies have described the Possum microbiota, however, one study found a prevalence of *Helicobacter* sp. in possums [48], which we also observed in the possum cloaca.

The results of this study have provided the first insight into the microbiota of northern quoll populations and a foundation for future microbiota studies. For example, future studies may consider the impact of reduced genetic diversity of the populations we have assessed on the diversity of the microbiota [49]. Understanding the relationship between the microbiota of quoll populations will undoubtedly increase our knowledge surrounding the health and biology of these marsupials. Subsequently, conservation efforts will be enhanced by the ability to monitor the health of translocated or reintroduced populations in the future.

## Figures and Tables

**Table 1:** Primers used for 16S rRNA gene library preparation. Primer names are indicated in the first column, and different regions of the primers are separated into columns for ease of viewing.

***Primer Name***	***Illumina Adaptor Region***	***Phaser***	***Priming Region***
PCR1_forward	GTGACTGGAGTTCAGACGTGTGCTCTTCCGATCT	(0–3 bp)	ACTCCTACGGGAGGCAGCAG
PCR1_reverse	ACACTCTTTCCCTACACGACGCTCTTCCGATCT	(0–3 bp)	GGACTACHVGGGTWTCTAAT
	***Illumina Flowcell Region***	***Sample Index***	***Illumina Adaptor Region***
PCR2_forward	CAAGCAGAAGACGGCATACGAGAT	(8 bp index)	GTGACTGGAGTTCAGACGTG
PCR2_reverse	AATGATACGGCGACCACCGAGATCT	(8 bp index)	ACACTCTTTCCCTACACGA

## Supplementary Materials

The following are available online at http://www.mdpi.com/2076-2607/6/3/68/s1.
