# Supplementary material for: Cloacal and Ocular Microbiota of the Endangered Australian Northern Quoll
Source: Microorganisms. 2018 Jul 12;6(3):68. doi: 10.3390/microorganisms6030068 (PMC6163277; doi:10.3390/microorganisms6030068)
Supplement: Supplementary file 1 [file microorganisms-06-00068-s001.docx]

***PCR amplification of 16S rRNA gene libraries.***

PCR reactions for the first stage consisted of 1X KAPA HiFi HotStart Mix and 500nM forward and reverse primer (Table S1) and either 2ng DNA template, or the maximum volume of sample (20μL) where the DNA concentration was less than 10 ng/μl.  The total reaction volume was 50 μl. Cycling conditions were; initial denaturing at 95°C for 3 mins, 20 cycles of denaturation (15 seconds at 95°C); annealing (15 seconds at 50°C) and extension (30 seconds at 72°C) followed by a final extension at 72°C for 5 minutes.

The amplified products were purified using a PCR-mag magnetic clean-up kit  (Axygen) using a 0.8 X volume of magnetic beads to PCR reaction, followed by mixing and incubation for five minutes at room temperature. The solution was incubated on a 96 well magnetic plate (Beckman Coulter catalogue number A32782) for three minutes, after which the supernatant was removed. The beads were washed with 185 µL of freshly prepared 85% ethanol for 30 seconds. Residual ethanol was allowed to air dry for 5 minutes. Beads were resuspended in 31µL of ultrapure distilled water (Life Technologies catalogue number 10977023) and incubated for 5 minutes, followed by four minute incubation on the magnetic plate. The solution containing the DNA amplicons was removed and used as a template for the second stage PCR.

The second stage enrichment PCR reactions consisted of 1X KAPA HiFi HotStart Mix 250nM enrichment forward and reverse primers (Table S1) and the maximum amount of cleaned amplicon product from PCR Stage One (27.5µL) for a total 50µL volume. Cycling conditions were; initial denaturation at 95°C for three minutes; 10 cycles of denaturation (15 seconds at 95°C); annealing (15 seconds at 60°C) and extension (30 seconds at 72°C) followed by a final extension step (five minutes at 72°C).

To quantify the yield and success of the PCR amplification, 10µL of PCR product was diluted with EZ-VISION (Amresco catalogue number N313) in 6X loading buffer and analysed on a 1.5% agarose (Sigma-Aldrich A9539) gel prepared in 1X TBE buffer. For quantification, a 100bp ladder (New England BioLabs catalogue number N3231L) was also included to confirm the size of the PCR product.
